# Study of Biological Behavior and Antimicrobial Properties of Cerium Oxide Nanoparticles

**DOI:** 10.3390/pharmaceutics15102509

**Published:** 2023-10-23

**Authors:** Iason Chatzimentor, Ioannis Tsamesidis, Maria-Eleni Ioannou, Georgia K. Pouroutzidou, Anastasia Beketova, Veronica Giourieva, Rigini Papi, Eleana Kontonasaki

**Affiliations:** 1 Department of Prosthodontics, Faculty of Health Sciences, School of Dentistry, Aristotle University of Thessaloniki, 54124 Thessaloniki, Greece; chatzimentor@dent.auth.gr (I.C.); johntsame@gmail.com (I.T.); mioann1@dent.auth.gr (M.-E.I.); gpourout@physics.auth.gr (G.K.P.); anastasiabeketova@yahoo.com (A.B.); 2 Laboratory of Advanced Materials and Devices (AMDeLab), Faculty of Sciences, School of Physics, Aristotle University of Thessaloniki, 54124 Thessaloniki, Greece; 3 Laboratory of Biochemistry, Department of Chemistry, Aristotle University of Thessaloniki, 54124 Thessaloniki, Greece; gouvero@chem.auth.gr (V.G.); rigini@chem.auth.gr (R.P.)

**Keywords:** cerium oxide, nanoparticles, periodontal ligament cells, reactive oxygen species, osteogenic differentiation

## Abstract

(1) Background: An element that has gained much attention in industrial and biomedical fields is Cerium (Ce). CeO_2_ nanoparticles have been proven to be promising regarding their different biomedical applications for the control of infection and inflammation. The aim of the present study was to investigate the biological properties and antimicrobial behavior of cerium oxide (CeO_2_) nanoparticles (NPs). (2) Methods: The investigation of the NPs’ biocompatibility with human periodontal ligament cells (hPDLCs) was evaluated via the MTT assay. Measurement of alkaline phosphatase (ALP) levels and alizarine red staining (ARS) were used as markers in the investigation of CeO_2_ NPs’ capacity to induce the osteogenic differentiation of hPDLCs. Induced inflammatory stress conditions were applied to hPDLCs with H_2_O_2_ to estimate the influence of CeO_2_ NPs on the viability of cells under these conditions, as well as to reveal any ROS scavenging properties. Total antioxidant capacity (TAC) of cell lysates with NPs was also investigated. Finally, the macro broth dilution method was the method of choice for checking the antibacterial capacity of CeO_2_ against the anaerobic pathogens *Porphyromonas gingivalis* and *Prevotella intermedia*. (3) Results: Cell viability assay indicated that hPDLCs increase their proliferation rate in a time-dependent manner in the presence of CeO_2_ NPs. ALP and ARS measurements showed that CeO_2_ NPs can promote the osteogenic differentiation of hPDLCs. In addition, the MTT assay and ROS determination demonstrated some interesting results concerning the viability of cells under oxidative stress conditions and, respectively, the capability of NPs to decrease free radical levels over the course of time. Antimicrobial toxicity was observed mainly against *P. gingivalis*. (4) Conclusions: CeO_2_ NPs could provide an excellent choice for use in clinical practices as they could prohibit bacterial proliferation and control inflammatory conditions.

## 1. Introduction

Periodontitis is one of the most prevalent dental diseases that affects millions of people. It is a multifactorial inflammatory condition that affects the tooth-supporting apparatus (gingiva, periodontal ligament, cementum, and alveolar bone). Increasing concentrations of pathogenic bacteria in dental plaque and the formation of dental biofilm trigger a strong inflammatory immune response and are key factors in the outbreak and advancement of periodontal disease. One of the main initiating factors of chronic periodontitis is an imbalance of the microorganisms in the dental plaque. Additionally, altered dynamic interactions between subgingival microorganisms, host immune responses, risky environmental exposure, and genetic characteristics are linked to periodontitis and are likely its primary cause [1,2]. 

Dental implants constitute an integral part of the supportive phase of periodontal treatment when permanent teeth have finally fallen out. Peri-implant mucositis and peri-implantitis are the main biological complications concerning implant prosthodontic treatment. The composition of the microbial plaque preexisting at the time of implant placement largely determines the composition of the microbial flora on the implant’s surface [3], hence patients with a history of periodontitis are more likely to develop peri-implantitis as well [4]. The prevention of bacterial adhesion on the implant surface, the stability of osseointegration, and a decrease in inflammation surrounding the implants are three essential elements required to guarantee the long-term clinical success of dental implant restorations. To increase their lifespan, it is therefore desirable to create unique surface modifications for dental implants that have antibacterial and anti-inflammatory properties [5]. Implant surface modifications have recently been investigated in efforts to increase the amount of bone that contacts the implant, simulate the cellular environment, and facilitate the osseointegration mechanism. Nanotextured titanium, hydroxyapatite, or pharmacological compounds like bisphosphonates may be applied to coat implants to initiate and stimulate cellular differentiation and proliferation. Moreover, the idea of creating bioactive antimicrobial implant surfaces has been investigated. With a 90% reduction in viable bacteria within 2 min of UV radiation, nanostructured crystalline titanium dioxide coatings produced by cathodic arc have demonstrated bactericidal effects against *Staphylococcus epidermidis*. Silver nitrate-loaded nanotitanium surfaces and silver nanoparticle-modified titanium (TinAg) surfaces have both shown similar antibacterial effects [6]. Their clinical application may reduce the frequency of postoperative peri-implant infections and enhance effective treatments as silver nanoparticles have a broad spectrum of antibacterial potential.

The use of nanoparticles in the prevention of peri-implant inflammation and peri-implantitis has already been applied with the use of Ag and ZnO nanoparticles, as it is known that they possess antimicrobial and anti-inflammatory properties. However, their function was poor due to the cytotoxicity that restricts their usage [7,8]. Nowadays, Cerium (Ce) has garnered significant interest regarding the development of implants with antimicrobial and anti-inflammatory capabilities, as well as for facilitating osseointegration [9]. Ce is a rare-earth metal which is included in the lanthanide series of the periodic table [10]. It is found in bulk in two different redox states and dual oxidation modes (Ce^3+^ and Ce^4+^), leading to the creation of the oxides cerium dioxide (CeO_2_) and cerium sesquioxide (Ce_2_O_3_) [11,12]. Cerium oxide nanoparticles (CeO_2_ NPs), frequently referred to as nanoceria, have been used for years in glass polishing and chemo-mechanical polishing applications [13]. It should be noted that when particle diameter decreases, the number of Ce^3+^ sites increases, causing oxygen vacancies to disappear from the surface of CeO_2_ NPs [14]. CeO_2_ NPs are capable of performing free radical scavenging, radiation protection, and oxidative stress attenuation due to the Ce^3+^/Ce^4+^ redox cycle. From the perspective of tissue engineering, they can offer notable biological functions to facilitate tissue repair and regeneration, including antioxidant, anti-inflammatory, antibacterial, angiogenic, and antiapoptotic functions [15,16]. CeO_2_ NPs are characterized by numerous defects in their surface which are correlated to the oxygen vacancies at the nanoparticle lattice’s surface, and which result in the autocatalytic features of nanoceria. In addition, an increase in the surface–volume ratio of nanoceria shows a relationship with an increase in oxygen exchange and its redox reactions. There is a larger concentration of Ce^3+^ and oxygen vacancies on the surface of nanoceria than in the remainder of their volume, which means increased Ce^3+^ and redox potential. Hence, it is easier for the oxygen defects to be formed at nanoscale. In this manner, cerium oxide nanoparticles obtain the ability of free radical scavenging, by swapping between Ce^3+^ and Ce^4+^ redox states continuously [17,18,19].

The noteworthy advantages of CeO_2_ NPs give credence to the idea that they can be effective for altering bone biomaterials and encouraging immunomodulation, which would enable the regulation of macrophage behavior, and encourage stem cell osteogenic differentiation and bone tissue repair [20]. CeO_2_ NPs have been used in bone tissue engineering because they can regulate mesenchymal stem cell (MSC) development and differentiation, encourage bone regeneration on titanium surfaces, and improve vascularization [20,21]. Their usage in clinical practice could be as different surface coating techniques for metal oxide NPs, such as dip coating, spray coating, and spin coating [22]. Apart from the bone regenerative abilities and the role of CeO_2_ NPs as osteogenic agents, there are absolute indications that nanoceria could provide a prerequisite for successful regeneration with antioxidant, anti-inflammatory and antimicrobial factors [23]. As has already been described, nanoceria possesses an auto-regenerative cycle with interchanges between two redox states that aids in offering similar action to antioxidant enzymes and scavenging ROS. The contribution of CeO_2_ NPs to the anti-inflammatory outcome of engineered tissues is attributed to their ability to scavenge reactive species, reduce inflammation, decrease cytokine levels, and provide cell protection in vitro and in vivo.

The fundamental basis of the antibacterial behavior of NPs is their interaction with the bacterial cell membrane [24]. CeO_2_ NPs do not penetrate the cell; however, by inducing oxidative stress with the production of ROS, antibacterial activity is expressed. ROS create a chemical degradation of the organic constituents of microorganisms, like DNA and RNA, and also of their proteins. The nature of the reversible conversion of Ce^3+^ to Ce^4+^ is the reason for ROS generation. When the CeO_2_ atoms meet the bacterial cell membrane, this reduction takes place on the membrane and activates an intense biological procedure that induces cell death immediately after direct contact [25,26]. Furthermore, CeO_2_ NPs demonstrate their antibacterial potential by their nutrient support prevention, as they may intercommunicate with mesosomes and disrupt cellular respiration, DNA replication, and cell division when they adsorb onto the interface of bacterial cell walls. They may also augment the surface area of bacterial membranes [27]. The indirect interaction of CeO_2_ NPs with the bacteria has an additional role in the antimicrobial effect. CeO_2_ NPs combine with the ions or ROS of the intercellular space and harm bacteria by shifting these ions from the surface of the nanoparticles to the bacterial cell membrane [28].

The selected cell lineage in this research was hPDLCs. The PDL is an active and specialized connective tissue that develops from neural crest cells in the dental follicle [29]. PDLCs are made up of a variety of cell types, including fibroblasts, endothelial cells, epithelial cell rests of Malassez, sensory cells, osteoblasts, and cementoblasts [30]. According to Li et al. [30], hPDLCs possess stem cell properties and are capable of differentiating the osteoblastic lineage under different circumstances. Furthermore, due to their promising osteogenic differentiation ability, high proliferation rate, accessibility, and abundance, hPDLCs have garnered significant interest as a mesenchymal stem cell source [30,31]. The ability of hPDLCs to express high regenerative potential and to act as a source of cells for regeneration was the main reason they were chosen for the present study [30].

The primary aim of the present study was the investigation of the in vitro biological behavior of CeO_2_ NPs in hPDLCs and their antibacterial properties against common periopathogenic bacteria. Specifically, the purpose of the experimental procedures was to evaluate the lack of cytotoxicity of NPs, and the promotion of proliferation of hPDLCs under normal and oxidative stress conditions. Furthermore, CeO_2_ NPs were tested for the promotion of osteogenic differentiation of cells, successful ROS scavenging capacity, and their antibacterial properties against *Porphyromonas gingival* is (*PG*) and *Prevotella intermedia* (*PI*).

## 2. Materials and Methods

### 2.1. Synthesis of Cerium Oxide NP

All preparations were based on the Sol-Gel technique, with certain modifications [32]. Each CeO_2_ NP was created by dissolving 0.2 g of gelatin in 20 mL of double-distilled water at 40 °C and agitating the mixture with a magnetic stirrer until it became clear. After the gelatin had dissolved, the gradual addition of different quantities of cerium nitrate hexahydrate (Ce(NO_3_)_3_•6H_2_O^−^) ranging from 1 g to 5 g resulted in the production of five distinct samples [33]. The solution was rapidly agitated for an additional 30 min after the cerium precursor had been added before adding the amount of ammonia solution drop-by-drop until the pH reached 10. The solution turned from light yellow to yellow as the pH was increasing. The components were mechanically stirred for an hour after the addition of ammonia. The resulting mixture was centrifuged at 5000 rpm for 3 min before being repeatedly rinsed in acetone and water. The sample was then heated at 80 °C for 12 h in order to move to the drying stage of the production of CeO_2_ NPs, and a dried gel was produced. The synthesized matter was mashed until a powdery substance was obtained. The dried gel was then sealed in platinum capsules and calcinated in a high-temperature furnace for one hour at 550 °C (1 °C/min). Comprehensive characterization was conducted by employing FTIR, XRD, SEM, and TEM analyses. Morphological attributes and size distribution were ascertained through TEM, while selected area electron diffraction and high-resolution TEM confirmed the presence of a cubic CeO_2_ fluorite structure. Notably, the Ce concentration with the smallest particles exhibited a mean diameter of 10 nm (Supplementary Materials).

### 2.2. Establishment of Primary Cultures

Human biopsies of periodontal ligament tissues from a healthy donor, collected during a regular third molar extraction, were used to create hPDLCs cultures. In tissue culture flasks containing 5 mL of DMEM, 10% fetal bovine serum (FBS, Invitrogen, Waltham, MA, USA) and antibiotics (100 U/mL medium of penicillin, 100 mg/mL streptomycin, Invitrogen), small fragments of tissues created by mincing were deposited. The cultures were preserved at 37 °C in an incubator with an atmosphere of 95% humidity and 5%. After obtaining a significant fibroblast expansion (80% confluence), the cells were trypsinized with 0.25% trypsin/1 mM EDTA and were then cultivated in 24-well plates under normal conditions. The hPDLCs had spindle-like shapes and elongated morphology. The Institutional Ethical Committee approved the project (#110/10-2-2021).

### 2.3. Cytotoxicity Measurement of NPs

The study of the viability and the cytotoxicity of CeO_2_ NPs mixed with the hPDLCs was performed through the MTT[3-(4,5-dimethylthiazol-2-yl)-2,5-diphenyltetrazolium bromide] assay. The nanoparticles were evaluated at different concentrations (C_1_ = 0.125 mg, C_2_ = 0.25 mg, and C_3_ = 0.5 mg). The samples were added into the test tubes and 30 min of UV light was used for sterilization. After UV sterilization, 10 mL of DMEM was added into each test tube with the NPs. Then, 10^3^ cells per well were seeded overnight in 96-well plates for 24 h in a 37 °C sterile incubator. The next day, the NPs were added in triplicates and incubated for 24, 72, and 120 h in a sterile incubator at 37 °C with 5% CO_2_. Negative control cells were cultured with DMEM, and positive control cells were cultured with conventional medium (DMEM, fetal bovine serum 10%, and Penicillin/Streptomycin (P/S) 1%). Control groups were tested in triplicates. After each time point, MTT solution was added for 3 h. Following this procedure, the supernatants were separated and DMSO was added to dissolve formazan crystals for 30 min. After that time, the plate was placed in a microplate reader and the absorbance was measured spectrophotometrically at 540 and 630 nm.

### 2.4. Osteogenic Differentiation

Osteogenic differentiation testing was employed for the 5 g CeO_2_ NPs. The sample was sterilized for 30 min under UV light at two different concentrations (C_1_ = 0.125 mg and C_2_ = 0.5 mg) and then seeded with the hPDLCs. Cells with number of 4 × 10^4^ were seeded onto 12-well plates 24 h before the experiment. Osteogenic medium (OM) was used for the differentiation of hPDLCs, which contained complete culture medium-CCM (α-minimum essential media (α-MEM) (PAN BIOTECHGmbH, Aidenbach, Germany); 10% FBS (BIOWEST, Nuaillé, France); and antibiotics) enhanced with 0.01 µM dexamethasone (Cayman Chemical Company, Ann Arbor, MI, USA); 50 µg/mL L-ascorbic acid 2-phosphate (Cayman Chemical Company, MI, USA); and 10 mM sodium β–glycerophosphate (Cayman Chemical Company, MI, USA) [34]. The experiment was performed with the following groups: (1) cells seeded with NPs in OM, (2) cells seeded with NPs in conventional medium (3) as a positive control, cells seeded without NPs in OM, and (4) cells without NPs in CCM as a negative control. The experiment was executed at two time points (14 and 21 days), with the OM and CCM being changed every 2 days. The impact of CeO_2_ NPs on the osteogenic differentiation of hPDLCs was evaluated through alkaline phosphatase (ALP) activity and alizarin red staining (ARS).

#### 2.4.1. Alkaline Phosphatase Activity

ALP levels of hPDLCs were evaluated at 14 and 21 days with analysis of both cell lysates and their supernatants. Tris-HCl 25 mM, TritonX-100 0.5% was used to lyse the cell membranes. In detail, 80 μL of the lysate or supernatant was used for the ALP assays and 1.5 M 2-amino-2-methyl-1-propanol (pH 10.3) basic buffer was placed in each well to initiate the reaction. Consequently, a substrate solution, made by mixing 100 mg of 4-nitrophenyl phosphate disodium salt hexahydrate in 25 mL of ddH_2_O, was added at a concentration of 100 μL per well and kept for one hour at 37 °C before measuring the absorption at 405 nm.

#### 2.4.2. Alizarine Red Staining (ARS)

ARS is a pigment that shows the matrix mineralization by binding specifically to calcium salts. The cells were incubated in a 24-well plate. The 5 g sample of CeO_2_ NPs was tested, in combination with 4 × 10^4^ hPDLCs, at two different concentrations (C_1_ = 0.125 mg and C_2_ = 0.5 mg) after 14 and 21 days. CCM and OM were used as culture mediums. Cells cultured with OM and CCM served as control groups. A medium change was performed every two days. After the 14 and 21 days period of cell seeding with NPs, the supernatant was removed from the well-plates and the cells were washed out with PBS. Afterward, the fixing of cells with 70% ethanol was held for 1 h, followed by washing out with 500 μL PBS and staining with Alizarine red stain (Sigma-Aldrich, St. Louis, MO, USA) for 30 min. The plates were washed out again with distilled water and images were captured. After the 30 min period, the dye was eluted from the cells with 10% cetylpyridinium chloride for 1 h and the absorbance was measured with the microplate reader (PerkinElmer, Waltham, MA, USA) at 540 nm. ARS was used to assess whether areas of created and deposited mineralized/calcified matrix were present [34]. For calculating the OD values obtained from the NPs alone, without cells, an identical set of assays was carried out with only NPs, without hPDLCs [35].

### 2.5. Effect of CeO_2_ NPs on Stressed hPDLCs

#### 2.5.1. MTT Assay

The ability of CeO_2_ NPs to encourage cell survival in induced oxidative stress conditions was measured again by MTT assay. The goal of the experiment was to assess the ability of CeO_2_ NPs to promote cell viability in cell cultures where oxidative stress had been induced, as well as to control the levels of the H_2_O_2_ of the already-stressed cells. H_2_O_2_ has been used previously as a means to simulate periodontal inflammation [36]. For that purpose, the oxidative stress markers in hPDLCs upon direct interaction with CeO_2_ NPs before and after H_2_O_2_ application were measured. Further, 10^3^ cells/well were left to attach in a 96-well plate and incubated for 24 h in a sterile incubator at 37 °C. The following step was the addition of H_2_O_2_ in the amount of 125 μM, which was reached after a series of dilutions, for 24 h as a non-toxic concentration for the survival of the cells. As control group cells cultured in CCM were used, the oxidative stress-induced cells (treated with H_2_O_2_) without NPs were also tested at every incubation time point. The next day, the different CeO_2_ NPs were added (1 g was excluded based on suboptimal structural properties), after the removal of all medium conditions (except oxidative stressed-induced cells treated with H_2_O_2_ without nanoparticles), in triplicates at three different concentrations (C_1_ = 0.125 mg/mL, C_2_ = 0.25 mg/mL, and C_3_ = 0.5 mg/mL), exactly as in the MTT assay for the hPDLCs without oxidative stress induction.

#### 2.5.2. Investigation of ROS Levels in H_2_O_2_ Stressed hPDLCs

Cell-permeable ROS-sensitive probe 2′,7′-dichlorodihydrofluorescein diacetate (CM-H_2_DCFDA), which fluoresces at 520 nm (ex = 480 nm) after oxidation, was used to appraise the levels of intracellular ROS [37]. Τo induce inflammatory conditions without inducing the death of the hPDLCs, 125 μΜ H_2_O_2_ was applied for 24 h as a pretreatment to increase ROS generation from the hPDLCs [38]. For the preparation of the preconditioning solution, consecutive dilutions of 30% H_2_O_2_ with DMEM were performed in order to achieve the quantity of 125 μM in 200 μL per well. The following day, cells were observed in the optical microscope and mixed with the NPs. For each of the 4 different NPs samples examined, three different concentrations were used (C_1_ = 0.125 mg/mL, C_2_ = 0.25 mg/mL, and C_3_ = 0.5 mg/mL) in triplicates and the hPDLCs were incubated in the same way and time points as in the cytotoxicity assay (37 °C, 95% humidity and 5% CO_2_ atmosphere). Cells in the culture medium without exposure to H_2_O_2_ and cells exposed to H_2_O_2_ were used as control groups. After the incubation period, the supernatant of each well plate was transferred to another 96-well plate to be stored and the cells were left in the initial plate for cell lysis. The washing out of the cells with PBS solution was performed, followed by the addition of 200 μL of the lysis buffer (TrisHCl 25 mM, TritonX-100 0.5%) at 4 °C for 2 h. In order to calculate the maximal emission, 3 mM H_2_O_2_ were added, and fluorescence was resolved with the operating system “Xenius”. The levels of ROS at different time points of the incubation of NPs were evaluated by measuring the fluorescence of hPDLCs suspensions using 20 μΜ of CM-H_2_DCFDA in 96-well black-walled microplates (Corning^®^, Sigma Aldrich, St. Louis, MO, USA). The relative fluorescence was measured with a Tecan fluorometer and is expressed as “% maximal emission”, where maximal emission was defined as the fluorescence emission obtained following the addition of 3 mM H_2_O_2_. hPDLCs were seeded in 96-well plates in triplicates, with a density of 10^3^ cells/well and DMEM as the culture medium. The cells were incubated for 24 h in a sterile autoclave.

### 2.6. Total Antioxidant Capacity Investigation

The same conditions described in the ROS investigation were followed to induce oxidative stress conditions, and TAC measurement was realized for the same concentrations of the 4 g and 5 g samples of CeO_2_ NPs. The total antioxidant capacity was investigated with a TAC kit (TAC colorimetric assay kit, Cayman Chemical Co., Ann Arbor, MI, USA) by applying the already-defined TEAC technique [39].

### 2.7. Antibacterial Activity

One of the most popular methods for determining the inhibitory capacity of antimicrobial agents, such as antibiotic compounds with bactericidal or bacteriostatic action, is broth dilution [40]. Using the macro broth dilution method, CeO_2_ NPs were tested for their antimicrobial susceptibility against two gram-negative anaerobic strains, *Prevotella intermedia* (DSM 20706) and *Porphyromonas gingivalis* (DSM 20709). Stocks of bacteria were frozen and kept at −80 °C. The strains were raised in the proper media, adjusted peptone yeast glucose (PYG) medium for *P. intermedia* and shredded meat medium with carbohydrates for *P. gingivalis*, for 3–4 days at 37 °C in anaerobic environments with 5% CO_2_ and agitation (130 rpm). Using a JENWAY 6305 spectrophotometer, optical density (OD_600_ nm) was used to measure the rate of bacterial growth. Five samples of CeO_2_ NPs were tested for antibacterial effectiveness at various concentrations (0.125, 0.25, 0.5, 1, 2 mg/mL). The nanoparticles were applied to a 10% (*v*/*v*) suspension for each bacterium (equivalent to 10^8^ CFU/mL), for 3 days (for both *P. gingivalis* and *P. intermedia*). Before seeding, the NPs were rapidly sonicated within each medium for 20 min. Consequently, an optical density of 600 nm was determined to define the maximum percentage of bacterial inhibition by CeO_2_ NPs. Duplicate runs of each experiment were completed. Additionally, blanks (medium with nanoparticles) and control growth (medium with and without inoculum) were assessed. The absorbance ratio of the treated bacteria suspension to the fully grown suspension (control) was used to calculate the percentage of absorbance.

### 2.8. Statistical Analysis

The variable of OD% was represented with mean and standard deviations. A two-way analysis of variance (ANOVA) was used to compare OD% across time and combinations of material/concentration for both bacteria, *P. gingivalis* and *P. intermedia*. Two-way repeated measures ANOVA was used to compare OD% across time and combinations of medium/material/concentration with the MTT-based method, ARS-based method, ALP-based method (both for lysates and supernatants), ΜΤΤ with H_2_O_2_ cell viability assay, and ROS level analysis. Bonferroni corrections were made to adjust for multiple comparisons. Statistical analysis was performed using IBM SPSS Statistics 28 (IBM Corp., Armonk, NY, USA). Statistical significance level was set at *p*-value < 0.05.

## 3. Results

### 3.1. Cytotoxicity Measurements of NPs

#### MTT Cytotoxicity Assay

**Figure 1:**
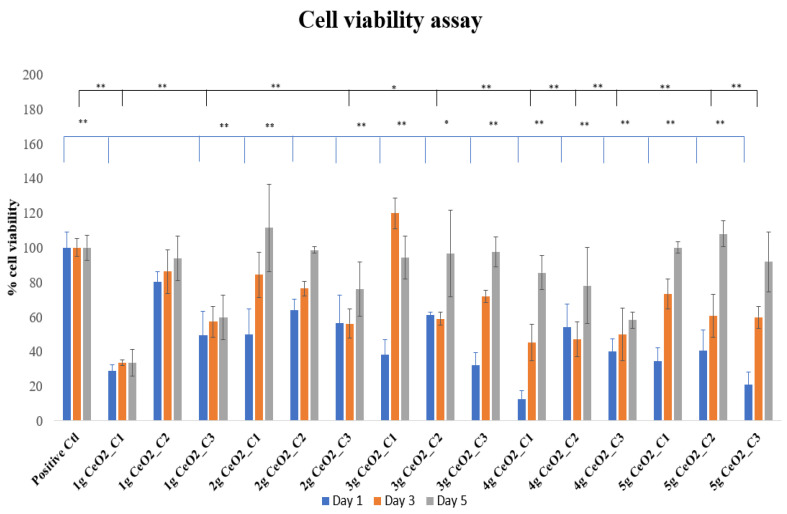
MTT cell viability assay of hPDLCs treated with various concentrations of CeO_2_ NPs. The bars show the statistically significant variations between cells alone and cells that were treated with NPs (* = *p* < 0.05, ** = *p* < 0.001).

The cytotoxicity assay results after the incubation of nanoparticles with hPDLCs for 1, 3, and 5 days are presented in Figure 1.

The tested materials were compared to the control groups. Specifically, it can be observed that on day 1, all concentrations of the NPs showed a decreased cell viability in comparison with the control group. Enhanced cell proliferation was noticed by day 3 and especially on day 5, which was indirectly confirmed by an increase in the optical density values compared to the control OD. The maximal increase in cell proliferation took place on day 3 for 3 g CeO_2_, where C_1_ increased cell proliferation by 20% compared to the control. Although there was an increase on day 3 compared to day 1 for most of the NPs and concentrations, the control cells presented a statistically significant increase in cell viability compared to specific combinations. On day 5, the percentage of optical density was above the 80% cell viability threshold in most of the CeO_2_-seeded hPDLCs cultures, with the 2 g, 3 g, and 5 g samples of CeO_2_ showing promising results concerning cell viability. The tendency of cells to increase their proliferation after day 1 supports the perspective that the NPs initially inhibit cell proliferation; but over the course of time, they increase the viability of hPDLCs, verifying a time-dependent behavior. There were no statistically significant differences between the control cells and the other combinations of material and concentration.

### 3.2. Osteogenic Differentiation

#### 3.2.1. Alizarine Red Staining

**Figure 2:**
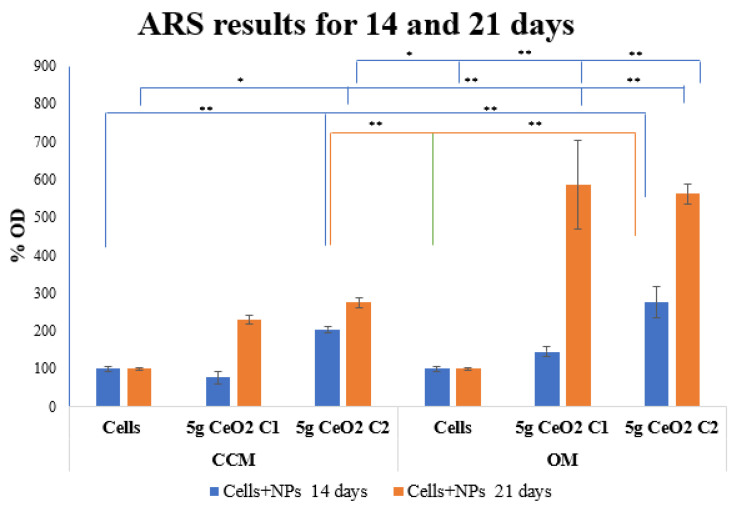
Alizarine red staining results of hPDLCs treated with different concentrations of 5 g CeO_2_ NPs at 14 and 21 days for CCM and OM. The bars show statistically significant variations between cells alone and cells that were treated with NPs (* = *p* < 0.05, ** = *p* < 0.001).

ARS was used to assess if mineralized matrix regions had been produced and deposited among the nanoparticles and the hPDLCs. As shown in Figure 2, the deposition at 14 days seems to show a statistically significant increase in comparison with the control groups, except for the C_1_ of CeO_2_ 5 g NPs in the conventional medium.

**Figure 3:**
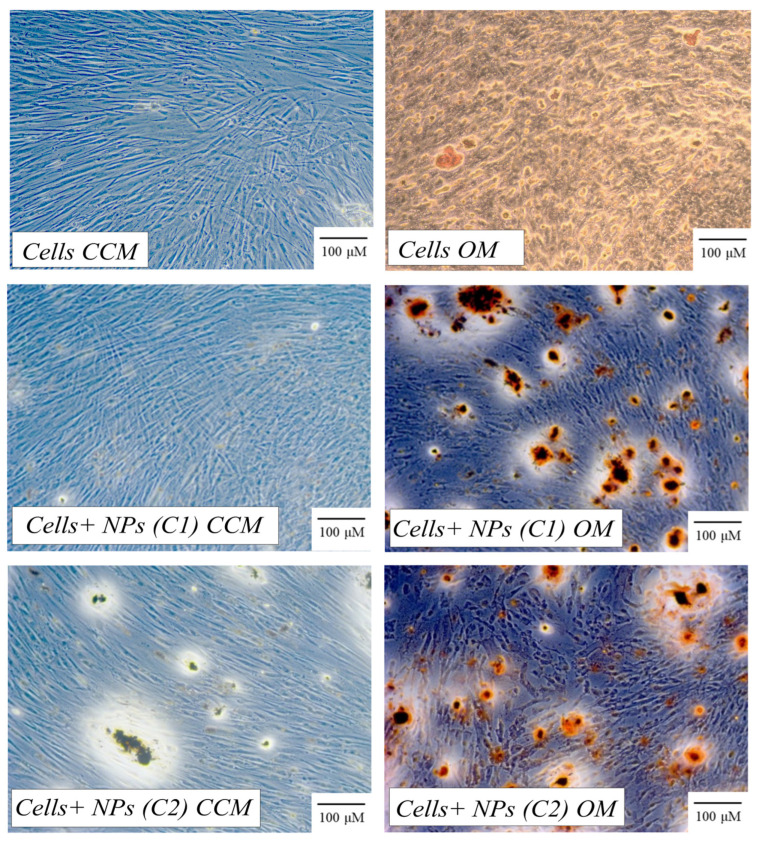
Pictures from in vitro calcium nodules deposition from hPDLCs seeded without or with CeO_2_ NPs at different concentrations and with different culture medium at 21 days.

The presence of nanoparticles in both CCM and OM resulted in a statistically significant increase in ARS staining at 14 days. It is worth noting that the nanoparticles significantly promoted the osteogenic differentiation of the hPDLCs. The most evident calcium salts deposition was on day 21 (Figure 3), where a massive increase in ARS was observed, especially for the NPs cultured with hPDLCs in OM. The seeding of hPDLCs with nanoparticles provided a statistically significant increase in the mineralization marker at 21 days in both CCM and OM. Regarding the biomineralization effect of NPs on the cells, there is a time-dependent relationship, which is observed by the excessive increase in OD percentage values at the 21-day time point.

#### 3.2.2. Alkaline Phosphatase Activity

**Figure 4:**
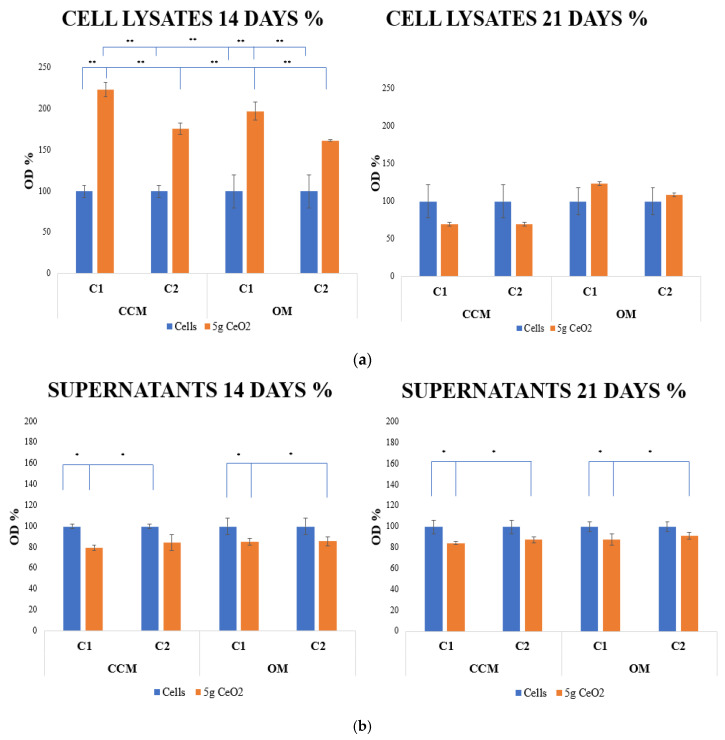
ALP activity results of hPDLCs cell lysates (**a**) and cell supernatants (**b**) treated with different concentrations of CeO_2_ NPs in CCM and OM. The bars show statistically significant variations between cells alone and cells that were treated with NPs (* = *p* < 0.05, ** = *p* < 0.001).

ALP activity was used to assess the effect of CeO_2_ NPs on hPDLCs osteogenic differentiation. The levels of ALP were measured on days 14 and 21 for both the cell lysates and supernatants of cultured hPDLCs with two different concentrations of 5 g CeO_2_ NPs. The activity of ALP for the hPDLCs cell lysates at 14 and 21 days is presented in Figure 4a. ALP activity levels presented statistically significant differences in comparison with the control groups on day 14, with an increase in the ALP value for both concentrations of NPs. On day 21, cell lysates also showed an increased expression of ALP in OM, whereas the results did not present statistical significance.

### 3.3. Effect of CeO_2_ NPs on Oxidative Stressed hPDLCs

#### 3.3.1. MTT Assay with H_2_O_2_

**Figure 5:**
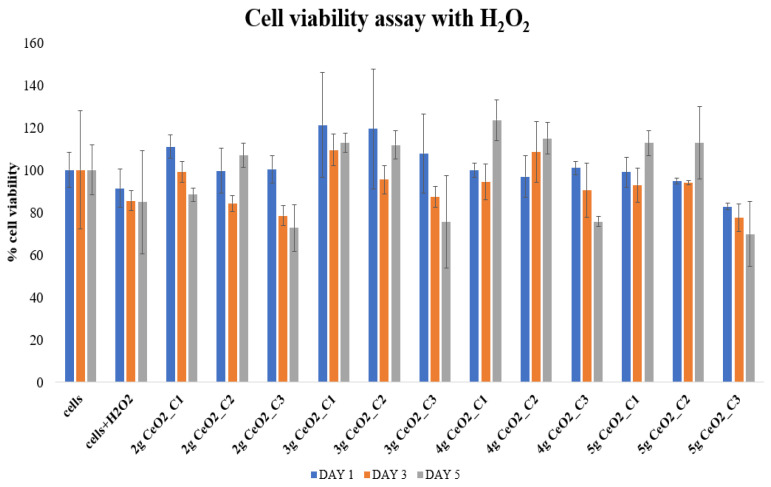
Cell viability assay of hPDLCs treated with various concentrations of CeO_2_ NPs, after exposure to 125 μΜ H_2_O_2_ to induce oxidative stress conditions and mimic inflammatory conditions without causing cell death [43].

Hydrogen peroxide (H_2_O_2_), which has been shown in other studies to be able to efficiently translocate cell membranes and create hydroxyl radicals, was used to recreate the injury of the hPDLCs by triggering oxidative stress to mimic periodontal disease inflammatory conditions [41,42]. After the incubation of stressed cells with CeO_2_ NPs at different concentrations, the results (Figure 5) indicated a general maintenance of cell viability in the presence of the NPs.

Specifically, on day 1, most of the cultures showed that cells are able to stay viable under these conditions, with the 3 g CeO_2_ NPs reaching a 121.2 ± 28.2% non-statistically significant increase in cell viability in comparison with the control group. However, there were no statistically significant differences between the NP-seeded hPDLCs and the control groups on day 1. Statistically, this same concept was maintained on day 3, while most of the NP concentrations were over the 80% cell viability level. Day 5 also indicated no statistically significant differences compared to the control groups, although some concentrations of 3, 4, and 5 g samples had statistically significant differences in relation to other samples and concentrations. In general, the incubation of nanoparticles contributed to the protection and survival of cells under oxidative stress conditions as the cells were able to survive under the presence of H_2_O_2_. The fact that incubated cells with H_2_O_2_ remained viable is a sign that the quantity of 125 μM was not able to introduce cell death, but only a mild stress.

#### 3.3.2. ROS Determination in Stressed hPDLCs

**Figure 6:**
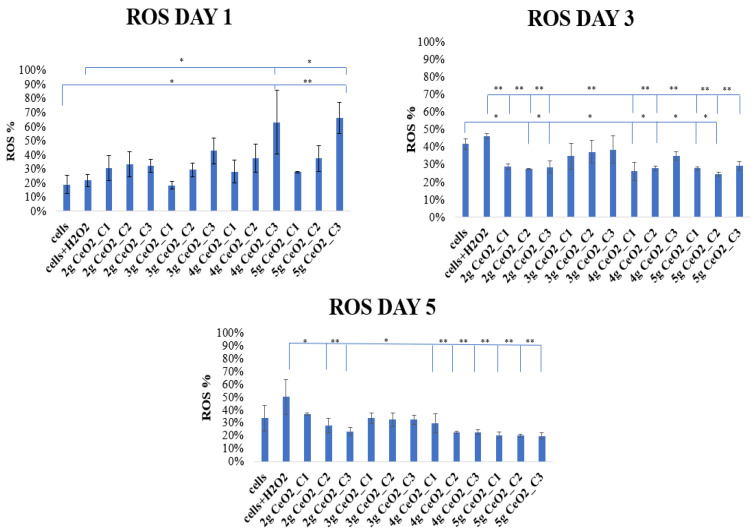
Intracellular ROS levels of hPDLCs at different time points after incubation with CeO_2_ NPs of different concentrations (C_1_ = 0.125 mg/mL, C_2_ = 0.25 mg/mL, and C_3_ = 0.5 mg/mL). The bars show statistically significant variations between cells alone and cells that were treated with NPs (* = *p* < 0.05, ** = *p* < 0.001).

Figure 6 shows the levels of intracellular ROS in pretreated hPDLCs with H_2_O_2_ upon incubation with various concentrations of CeO_2_ NPs for 1, 3, and 5 days. After the analysis of the results, an increased production of free radicals in NP-seeded hPDLCs on day 1 was observed in comparison with both control groups. In detail, the ROS levels were generally low with no significant differences compared to the control groups, except for CeO_2_ 4 g and 5 g, in 0.5 mg/mL, where a statistically significant increase in ROS production was detected and a 63 and 66% increase in free radical levels was determined, respectively. Regarding ROS production, it was noted that there was a dose-dependent moderate increase with the increasing concentration of nanoparticles. On days 3 and 5, a statistically significant reduction in free radicals until basal levels of ROS were observed. This indicated a time-dependent factor of the action of NPs as ROS scavengers. Furthermore, with increasing concentrations of NPs, a decrease in free radicals was monitored, observed on day 5.

### 3.4. Total Antioxidant Capacity Measurement

**Figure 7:**
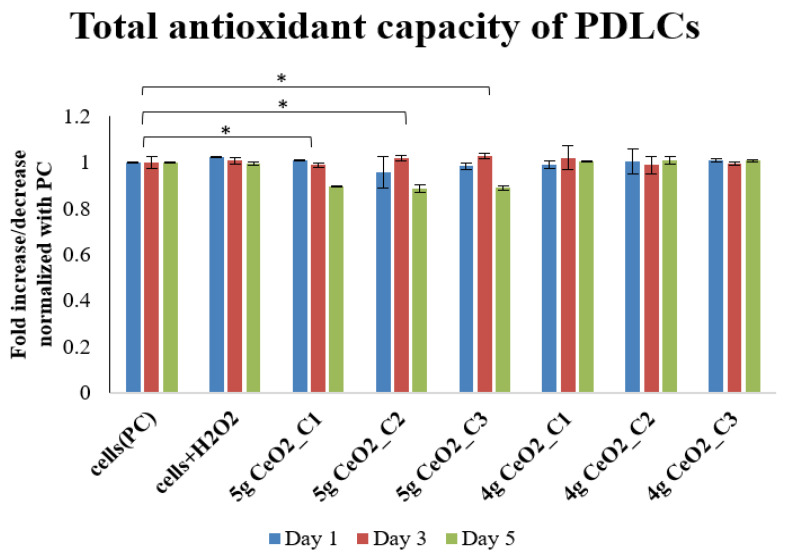
Total antioxidant capacity of hPDLCs cultured with two different samples of CeO_2_ NPs (4 g and 5 g) at different concentrations (C_1_ = 0.125, C_2_ = 0.25, C_3_ = 0.5 mg/mL). The results are in mM and are interpreted in fold adjustment compared to the control group (cells without NPs) (* = *p* < 0.05).

Once the ROS experiments concluded, the same set of experiments in cell lysates was performed for the evaluation of the antioxidant capacity of hPDLCs with selected NPs (4 g and 5 g) (Figure 7). On day 1 and 3 of incubation, the TAC assay specifically revealed that the addition of NPs (4 g and 5 g) had no effect on the antioxidant capacity of hPDLCs. The most pronounced differences were observed after 5 days of incubation with NPs. Specifically, the 5 g CeO_2_ group of NPs presented the lowest TAC capacity after 5 days of incubation, with low levels of ROS compared with both the cells alone and the cells under H_2_O_2_ stress. A decrease in TAC levels was present for all of the tested dosages (0.125, 0.25, 0.5 mg/mL) on day 5.

### 3.5. Antibacterial Activity of CeO_2_ NPs

**Figure 8:**
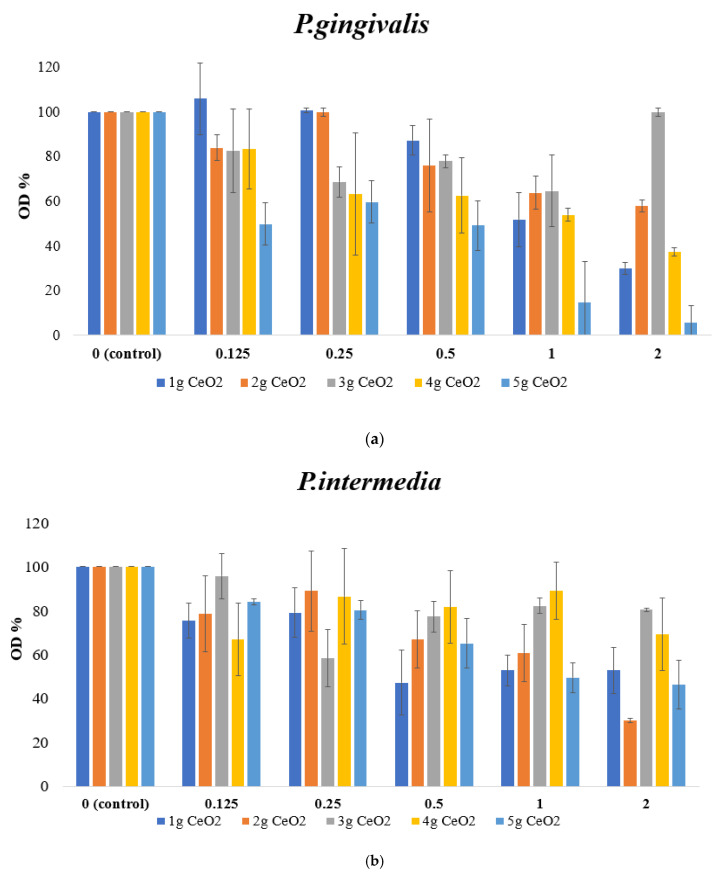
Antibacterial results of CeO_2_ NPs against (**a**) *Porphyromonas Gingivalis* and (**b**) *Prevotella intermedia*.

The antibacterial activity of all CeO_2_ NP samples was measured as % absorbance compared to the control. It seemed that there was a dose-dependent gradual decrease in bacterial proliferation as the concentration of CeO_2_ NPs increased for both bacterial groups. Generally, it appeared that most of the NPs had statistically significant lower percentages of bacterial populations, however, none of the concentrations were able to totally inhibit the bacterial growth. The NPs appeared to be more toxic against *P. gingivalis* (Figure 8a). Specifically, the 5 g CeO_2_ sample at the highest concentrations of 2 and 1 mg/mL was observed to be the most toxic against *P. Gingivalis*, as a statistically significant reduction of 94.6 ± 7.7% and 85.5 ± 18.6% in bacterial cell numbers was noticed in comparison with the respective control groups. On the other hand, *P. intermedia* appeared more tolerant to the CeO_2_ NPs. The highest percentage of bacterial cells inhibition was shown by the 2 g sample of CeO_2_ NPs, which, at the highest concentration of 2 mg/mL, diminished the number of bacterial strains by 70 ± 0.8% compared to the control group (Figure 8b). All the other groups of NPs showed a tendency to decrease their bacterial numbers, however none went below 45%.

## 4. Discussion

In this study, the biological properties and the antimicrobial behavior of CeO_2_ NPs was investigated. The results indicated that CeO_2_ NPs were non-toxic to hPDLCs, while concomitantly their presence increased the number of markers of osteogenic differentiation. In addition, it was detected that NPs were able to keep hPDLCs viable under oxidative stress conditions in the presence of H_2_O_2_ in cell cultures. The experimental procedures also verified that CeO_2_ NPs provide ROS-scavenging properties when seeded with the cells while the initial increase in free radicals can contribute to the antibacterial action of NPs, with the increasing ROS levels providing the substrate for a reduction in bacterial levels, especially for *P. gingivalis*.

As we already know from previous the studies of Kargozar et al. [44] and Ren et al. [45], CeO_2_ NPs possess excellent biological properties which are attributed to their two distinct redox states and their capability to form oxygen vacancies in their microstructure. Specifically, Kargozar et al. studied the physicochemical and biological properties of CeO_2_ NPs for potential use in tissue engineering and regenerative medicine [16]. The results of the previous study appear to have similarities with the study of Ren et al. in which the biological behavior and the osteogenic potential of hPDLCs was investigated in the presence of CeO_2_ NPs loaded on GTR membranes [45]. Additionally, due to their physicochemical properties, CeO_2_ NPs are able to inhibit the growth of bacterial species, such as drug-resistant pathogens like *Enterococcus faecium*, *Staphylococcus aureus*, *Klebsiella pneumoniae*, *Acinetobacter baumannii*, *Pseudomonas aeruginosa*, and *Enterobacter species* [26,46]. According to the results of our study, CeO_2_ NPs are non-toxic for hPDLCs and they can also promote cell proliferation. There is a time-dependent aspect of the cell viability and proliferation of hPDLCs seeded with nanoparticles, as indicated by the results of the MTT assay. Initially, the results from day 1 indicate the toxicity of NPs towards the cells, a result that is in contrast with the study of Tsamesidis et al. [23], in which artemisinin (ART)-loaded cerium-doped mesoporous calcium silicate nanopowders seem to present non-cytotoxic behavior on periodontal fibroblasts. However, on days 3 and 5, there was a progressive increase in the mitochondrial activity. Day 5 was the time point where the most pronounced increase in the percentage of cell proliferation was observed in the cells treated with NPs. This result is in accordance with the above study, where on day 5 there was a high level of cell proliferation of cerium-doped nanopowders.

Periodontitis is an inflammatory disease with bone loss as one of its most critical clinical characteristics. Usually, one of the targets of its treatment concerns the reconstruction of alveolar bone through regenerative procedures. It has already been mentioned that CeO_2_ NPs have a beneficial role in bone regeneration through their abilities to enhance the osteogenic differentiation [18]. In addition, according to Ho-Shui-Ling et al. [47], approaches to the treatment of bone regeneration could involve grafts, bioactive molecules, or a combination of cell therapies with or without bioactive molecules. In our study, the experiments showed some encouraging results concerning the potential of CeO_2_ NPs to promote the osteogenic differentiation of hPDLCs. Alizarine red staining was the first method used as a marker for the evaluation of the mineralization process by identifying calcium-containing nodule formations. The results of the experiment showed the great potential of nanoceria to increase the presence of mineralized matrix regions. At both time points, CeO_2_-seeded cultures increased their levels of osteogenic expression in comparison with the control group. It was interesting that on day 21, the presence of NPs provoked an impressive increase in osteogenic differentiation in the osteogenic medium, reaching levels more than 5.5 times higher than those of the control group. An identical concept is observed in the study of Ren et al., in which the osteogenic potential of CeO_2_ NP-loaded nanofibrous membranes seeded with hPDLCs was investigated. The results of their study similarly indicated an almost 3-fold increase in biomineralization in the presence of NPs at every concentration on day 21 [45]. Another study by Luo et al. examined the potential of CeO_2_ NPs in the promotion of osteoplastic precursor differentiation in MC3T3-E1 mouse osteogenic precursor cells. ARS was applied to investigate the rate of ECM mineralization. The results after 14 days were identical with the results of our study, where CeO_2_ NPs increased the expression of osteogenic markers in the osteogenic medium by 1.5 to 2 times. The same took place in our experiment, with results after 14 days indicating a similar increase in comparison with the control group for both concentrations of NPs [48].

The measurement of ALP levels was the second method used for the investigation of the osteogenic differentiation ability of CeO_2_ NPs. Alkaline phosphatase isozyme is widely expressed in bone-forming cells and is essential for the early stages of osteogenesis. It promotes cell maturation and calcification by hydrolyzing different kinds of phosphates. ALP is regarded as an early osteogenic differentiation marker [49]. According to Prins et al. [50], early stages of osteoblast engagement are characterized by a rise in ALP enzyme activity, and an elevation of ALP production throughout osteogenic differentiation is believed to reflect the proportion of osteogenic-oriented progenitor cells in a population. This approach agrees with the results of our study, as on day 14, we observed a statistically significant increase in the percentage expression of ALP activity in cell lysates, in both culture media and concentrations. However, on day 21, the results from cell lysates showed reduced ALP levels, with the exception of the NPs in OM where the ALP levels were higher than those of the control group. These results were predictable as this has been described by Stein and Lian [51]. ALP enzyme genes, along with other bone forming genes, are expressed during the proliferation period. ALP activity is up-regulated post-proliferatively, from days 7 to 16, when bone-forming cellular phenotypes can be revealed. During the maturation and mineralization period, ALP diminishes its activity. The extracellular matrix gradually takes on bone-like characteristics, and once mineralization begins, non-collagenous extracellular matrix proteins, such as osteocalcin, become more active and deposit inorganic calcium and phosphate crystals [52,53]. The same results were not observed with the cell supernatants, where the percentage of OD value was significantly below the levels of the control group in both days. The study by An et al., in which hPDLCs were cultured on three-dimensional biphasic calcium phosphate scaffolds, confirms that cell supernatants show an inhibition of ALP enzymatic activity in the presence of NPs [54].

The effect of CeO_2_ NPs was also tested under oxidative stress conditions to mimic periodontal inflammation. hPDLCs pretreated with H_2_O_2_ were used in combination with NPs and their effect on cell viability and ROS scavenging ability was tested. The experimental results of the MTT assay of stressed hPDLCs revealed a positive relationship between NPs and the promotion of cell survival and proliferation under inflammatory conditions. It was observed that on days 1 and 3, NPs helped to maintain cell viability over 80%, with the exception of the 2 g and 5 g samples at 0.5 mg/mL, where cell viability was at 78 and 77%, respectively. The results from day 5 indicated a dose-dependent aspect of the ability of NPs to control cell proliferation, as all NPs at their highest concentration of 0.5 mg/mL showed a decrease in mitochondrial activity in comparison with both control groups. Furthermore, NP-seeded hPDLCs seemed to increase their proliferation rate compared with the control groups, mainly on day 5. It is a fact that the concentration of 125 μΜ H_2_O_2_ did not appear to significantly reduce the viability of cells, as at every time point, they appeared to survive in these conditions. The results of our study showed similarities with the study by Fu et al. [38], where cAMP-responsive element-binding protein (Creb), extracellular signal-regulated kinase (ERK), and the apoptosis regulator Bcl-2 were the tested pathways for the protection of mouse periodontal ligament stem cells in the presence of different quantities of H_2_O_2_. Nouri et al. [43] proved that a lack of cytotoxicity from H_2_O_2_ is possible while they were studying the role of hypoxia-inducible factor-1 (HIF-1) against non-toxic concentrations of free radicals on MSCs. They examined different concentrations (10 μΜ, 20 μΜ, 50 μΜ, 100 μΜ) that can reduce cell viability in a dose-dependent manner after 12 h, however the levels of viable cells remained over or around 80% in all concentrations. Moreover, preconditioning of MSCs with non-toxic concentrations of 100 μΜ H_2_O_2_ with HIF-1 presence reflected positively on the survival of cells, although the mitochondrial activity was significantly lower than that of the non-pretreated cells with HIF-1. What is more interesting is that, in the results of our study, the presence of NPs offered the same results against free radicals as Creb and HIF-1 in the previous mentioned research. Additionally, there exists another experimental investigation by Da Costa et al. [36] where piceatannol, a metabolite extracted from plants, is reviewed as a potent antioxidant and anti-inflammatory defensive mechanism against oxidative stress. Piceatannol seems to be effective and counteract the free radicals produced by H_2_O_2_ by increasing the proliferation rate in human periodontal ligament fibroblast, similarly to the CeO_2_ NPs in our study.

CeO_2_ NPs were also tested for their potential activity as ROS scavengers. It is well-known that Ce, due to its physicochemical properties, acts against ROS by altering Ce^3+^ and Ce^4+^ redox states. The ROS-scavenging abilities of nanoceria are attributed to the presence of Ce^3+^ state [55]. As it has already been mentioned, the auto-regenerative cycle of CeO_2_ produces enzyme mimetic activity and categorizes it as a promising pharmacological agent [18]. In the present study, the potential ROS-scavenging properties of nanoceria were examined by measuring the levels of ROS after the induction of oxidative stress conditions by the exposure of the hPDLCs to H_2_O_2_. An H_2_-DCFDA ROS-sensitive probe, which emits bright green light after oxidation and is a great instrument for ROS detection, was used in this research [38]. The analysis was realized at three different time points by comparing NPs-seeded stressed hPDLCs with cells in conventional medium, as well as with H_2_O_2_-stressed cells. The results demonstrated an increase in free radicals on day 1, especially for the 4 g and 5 g samples, where a 63 and 66% increase in ROS levels was found, respectively. It is possible that these results correlate with the presence of antibacterial activity on day 1 in *P. gingivalis*, as the pro-oxidant activity of these NPs could enhance bacterial cell death. As is proposed by Alpaslan et al. [56], the NPs’ mechanism against bacteria is the generation of ROS, the interruption of their membrane, and the avoidance of reaching supplements. It is also notable that the presence of ROS after day 1 did not negatively influence the viability of the cells. As can be interpreted from the MTT results, hPDLCs incubated with NPs survived despite the presence of ROS, a result that proves the biocompatibility of CeO_2_ under these conditions. Likewise, on days 3 and 5, the ROS levels showed a time-dependent reduction to basal levels of 30% and less. The ROS-scavenging ability of the NPs was also dose-dependent, as was shown by the lowering levels of ROS as the concentration of NPs increased. The results of the antioxidant activity combined with the results from the ROS production indicate that the 5 g CeO_2_ NPs were able to modify the redox equilibrium of hPDLCs by reducing their antioxidant levels (TAC levels) and keeping ROS levels also low. These results suggest that a balancing act exists in the presence of NPs which prevents cell damage even at stress conditions. Nonetheless, this slight reduction in antioxidant capacity did not seem to negatively affect the in vitro deposition of the calcium process.

The antibacterial susceptibility of all CeO_2_ NPs was tested against *P. gingivalis* and *P. intermedia*. In the present study, the broth dilution method was the method of choice for testing the antimicrobial susceptibility of NPs against *P. gingivalis* and *P. intermedia*. This method uses liquid growth medium that is seeded with a specific number of bacterial cells and contains proportionally increasing quantities of the antimicrobial agent. When using a 2 mL amount for the test’s final volume, this process is referred to as macro-dilution [40]. Five different concentrations of CeO_2_ were tested for their antimicrobial susceptibility against the bacterial species. The results, after measurement of the absorbance at 600 nm, proved that the maximal concentration (2 mg/mL) of 5 g CeO_2_ NPs had the highest influence on the survival of *P. gingivalis*, with a reduction of 94.6 ± 7.7% in bacterial population. With increasing concentrations of NPs, *P. gingivalis* strains showed a proportional reduction in their population, however this reduction was only significant at the highest concentrations of 5 g CeO_2_. Similarly, a dose-dependent reduction also showed as the concentration of NPs was increased in *P. intermedia*. *P. intermedia* strains appeared even more tolerant, as a 70 ± 0.8% reduction in the bacterial population was observed at the highest concentration of the 2 g CeO_2_ sample. These results show the inhibitory effect of CeO_2_ NPs against bacterial growth. The antibacterial activity of CeO_2_ NPs has already been proven for gram-negative bacteria such as *P. gingivalis* and *F. nucleatum* in the study by Li et al., which investigated the antibacterial mechanism of nanoceria on the biofilm of titanium implants. Specifically, they show the capability of CeO_2_ to reduce the CFU/disk as well as the metabolic activity of *P. gingivalis* and *F. nucleatum* biofilms [5]. It is worth mentioning that the mechanism by which this reduction is possible is different from other metal oxide nanoparticles, such as TiO_2_, Ag_2_O, ZnO and CuO nanoparticles (NPs), which come into direct interaction with the bacterial cell wall to destroy it by releasing ROS [57]. On the other hand, as mentioned in the introduction, CeO_2_ NPs express their antibacterial characteristics through electrostatic interactions with the bacterial cell wall [28,58,59].

The present study was implemented with some limitations. In both MTT assays (with and without H_2_O_2_), the weighing of exact concentrations and the dispersion of NPs in order to avoid the creation of aggregates in the cultures was technically demanding. The ALP measurement of the experiment was not investigated on day 7, a time point during which it is possible that the levels of ALP enzyme would be significantly high. Furthermore, more concentrations of H_2_O_2_ could be used instead of only 125 μΜ in order to elucidate the protective role of NPs in cell viability and their ROS-scavenging ability at higher concentrations of oxidative stress. It would also be possible to maintain the presence of H_2_O_2_ in the hPDLCs cultures so that the ability of CeO_2_ NPs under continuous oxidative stress could be evaluated. Finally, different cell lines with respective properties to hPDLCs, like bone marrow stem cells (BMSCs) or periodontal ligament fibroblasts (PLFs), could be used instead.

## 5. Conclusions

The results of the study give rise to the conclusion that the presence of NPs, especially of 5 g samples of CeO_2_, increase the viability of hPDLCs over the course of time, verifying the lack of cytotoxicity and the time-dependent improvement in cell proliferation. Furthermore, 5 g CeO_2_ NPs were capable of promoting osteogenic differentiation of hPDLCs, indicated by an increased expression of ALP and ARS. Nanoceria presence also acted protectively towards the survival and maintenance of hPDLCs’ viability under oxidative stress conditions, as well as significantly restricting oxidative stress, and resulted in the production of basal ROS levels and/or ROS scavenging. Finally, the antibacterial potential of 5 g CeO_2_ NPs of the highest concentrations was verified, mainly for *P. gingivalis*.

## Data Availability

All data are presented in the manuscript.

## Supplementary Materials

The following supporting information can be downloaded at: https://www.mdpi.com/article/10.3390/pharmaceutics15102509/s1, Figure S1: TEM image illustrating the CeO_2_ nanoparticles recorder from samples 2 g CeO_2_, 3 g CeO_2_, and 4 g CeO_2_. The corresponding TEM images of the samples of 1 g CeO_2_ and 5 g CeO_2_ were previously published by our group. Table S1: mean particle size of the synthesized CeO_2_ nanoparticles.
