# Supplementary material for: Study of Biological Behavior and Antimicrobial Properties of Cerium Oxide Nanoparticles
Source: Pharmaceutics. 2023 Oct 23;15(10):2509. doi: 10.3390/pharmaceutics15102509 (PMC10610395; doi:10.3390/pharmaceutics15102509)
Supplement: Supplementary file 1 [file pharmaceutics-15-02509-s001.zip › pharmaceutics-2647842-supplementary.pdf]

Supplementary Materials

Characterization of Cerium Oxide Nanoparticles

Transmission electron microscopy (TEM)

The morphology and size dispersion were assessed through transmission electron microscopy (TEM), and Image J was employed to verify the diameters of the particles. Measurements were conducted on more than 100 nanoparticles, and a comprehensive record of their diameter distribution was maintained. TEM measurements were carried out using the FEI Tecnai G2 20 microscope operating at 200 kV. The procedure for crafting TEM samples encompassed the dispersion of the materials in ethanol, depositing them onto a carbon-coated grid, and subjecting it to a two-hour drying period.

Results of the Characterization of Cerium Oxide Nanoparticles

The microstructure of the CeO_2 nanoparticles at varying Ce concentrations, from the lowest (1gCeO_2) to the highest (5gCeO_2), was examined using TEM imaging techniques. Exemplary images captured from all specimens are presented in Figure S1. The NPs in the 1gCeO_2, 2gCeO_2, 3gCeO_2, and 4gCeO_2 samples display an almost spherical morphology, with dimensions spanning from 7.0 to 8.9 nm. In contrast, the 5gCeO_2 sample exhibited two distinct categories of crystalline nanoparticles in terms of size. It featured clusters with predominantly small, spherical nanoparticles around 13.3 nm, as well as those with irregular shapes and a significantly larger size compared to the rest of the samples, constituting the majority. The larger nanoparticles measured 35 nm (Table S1).

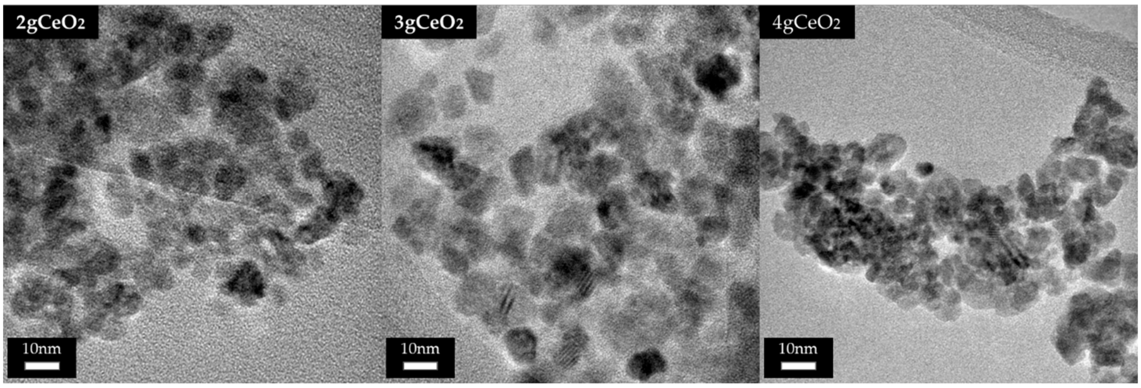

Figure S1: TEM image illustrating the CeO_2 nanoparticles recorder from samples 2gCeO_2, 3gCeO_2, and 4gCeO_2. The corresponding TEM images of samples 1gCeO_2 and 5gCeO_2 were previously published by our group[1].

Table S1: Mean Particle size of the synthesized CeO_2 nanoparticles

Sample	Particle size
1gCeO_2	7.0
2gCeO_2	7.5

3gCeO_2	8.9
4gCeO_2	7.1
5gCeO_2	13.3 & 35.0

References

Ioannou, M.E.; Pouroutzidou, G.K.; Chatzimentor, I.; Tsamesidis, I.; Florini, N.; Tsiaoussis, I.; Lymperaki, E.; Komninou, P.; Kontonasaki, E. Synthesis and Characterization of Cerium Oxide Nanoparticles: Effect of Cerium Precursor to Gelatin Ratio. *Appl. Sci.* **2023**, *13*, 2676. https://doi.org/10.3390/app13042676
